# Abbott’s Scissors

**Published:** 1884-01

**Authors:** 


### "ABBOTT'S SCISSORS."

Dr. Shepard, in a letter published in this number, points out the fact, one which had certainly escaped our observation, that the gum scissors illustrated in the November number have been in use in England for some time. Dr. Abbott says he devised the implement at the requisition of a prominent New York surgeon, and without any knowledge that such a thing existed. No one will accuse him of such a stupid and easily discoverable thing as the pirating of the invention. Even were he lacking in principle, he is certainly not devoid of sense. But if we are rightly informed the instrument is not even of original English invention. We have been assured that a description and illustration of a device, very like both Woodhouse's and Abbott's, was published in the *Missouri Journal* a number of years ago. We have not the complete files at hand to verify or disprove the assertion.

